# In this issue

**Published:** 2023-04

**Systematic review and pooled analysis of randomized controlled trials in Countries of the Gulf Cooperation Council (GCC).**

*Methods and quality assessment*

Alraddadi et al describe variations in characteristics of randomized controlled trials conducted in the Gulf Cooperation Council (GCC) countries, and critically appraising the quality of design, conduct and analysis of the trials. a systematically comprehensive electronic search of articles published between 1990 and 2018 and indexed in several databases were carried out. The proportion of adequately generated random sequence methods has increased yearly, and this increase accounted for a relatively large proportion over the latter half of the studied period, in contrast to the proportion of allocation concealment and blinding. The randomization methods have gained more attention over the last 3 decades

***see page 345***

**Figure:**
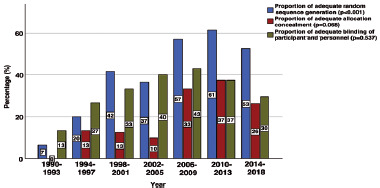
Quality of randomized-controlled trials published between 1990 and 2018.

