# Supplementary material for: In this issue
Source: Saudi Med J. 2023 Apr;44(4):327. (PMC10153616)
Supplement: Related texts

# Abstract: Original Articles

**The impact of viability assessment using cardiac MRI and echocardiogram on the outcome of revascularization in patients with multi-vessels coronary artery disease and moderate to severe ischemic cardiomyopathy**

Alzahrani et al investigate he influence of viability assessment in the management of patients with ischemic cardiomyopathy (ICM). All patients with ICM with moderate to severely reduced left ventricular ejection fraction (LVEF) who underwent viability assessment using cardiac magnetic resonance imaging (MRI) and echocardiogram as modalities of imaging were included. Posttreatment LVEF improved by 5% in the viable group compared to the nonviable group. Patients with viable myocardium had better outcomes in which LVEF significantly improved posttreatment.

***see page 373***

**Figure:** Left ventricular ejection fraction by treatment modality and viability.

**Potential barriers to the daily use of hearing aids in children**

Yousef et al identify factors affecting hearing aid usage of 59 children ranging from 6 months to 6 years. Patients with moderate to profound sensorineural hearing loss with complete data-logging information stored in the hearing aid programming file from January 2020 until June 2021 were included. The average daily hearing aid usage was 5.5 hour (h) after 3 months, 7 h after 6 months; and 8.7± 4.7 h as reported by parents. Patient age was positively correlated with data logging at 3 months and 6 months. We found that children’s age, gender, severity of hearing loss, residential location, and parents’ educational level had a significant effect on daily hearing aid usage.

***see page 406***
